# Radiology management of a ‘breast unit’ during COVID-19 pandemic: a single institution experience

**DOI:** 10.2217/fon-2020-0585

**Published:** 2020-12-02

**Authors:** Christine Salem, Marie-Ange Hajj, Hampig Kourié, Antoine Haddad, Abir Khaddage, Eliane Nasser Ayoub, Khalil Jabbour, Malak Moubarak, David Atallah

**Affiliations:** ^1^Department of Radiology, Faculty of Medicine, Saint Joseph University of Beirut, 116-5137, Beirut, Lebanon; ^2^Department of Oncology, Faculty of Medicine, Saint Joseph University of Beirut, 116-5137, Beirut, Lebanon; ^3^Department of Pathology, Faculty of Medicine, Saint Joseph University of Beirut, 116-5137, Beirut, Lebanon; ^4^Department of Anesthesiology, Faculty of Medicine, Saint Joseph University of Beirut, 116-5137, Beirut, Lebanon; ^5^Department of Gynecology & Obstetrics, Faculty of Medicine, Saint Joseph University of Beirut, 116-5137, Beirut, Lebanon

**Keywords:** biopsy, breast cancer, breast unit, COVID pandemic, PCR, radiology, safety, screening

## Abstract

**Aim:** To describe the activity in the ‘breast unit’ at the department of radiology during the coronavirus disease 19 lockdown in a university hospital treating coronovirus disease 19 patients in a Middle-Eastern developing country. **Materials:** This was a retrospective study conducted from March 9 until 11 May 2020, in the breast unit at the department of radiology of a central university hospital in a Middle-Eastern developing country. Data were collected from 205 patients visiting the breast unit during the lockdown period and compared with the activity in the same period in the previous year. **Results:** Reduction of the breast unit activity was estimated at 73%. In addition, 153 mammograms, 205 ultrasounds, and 16 breast MRIs were done. Indications for mammogram were screening (41.5%), follow-up (22%), clinical symptoms (20%) and breast cancer surveillance (16.5%). MRI was performed mostly for preoperative surgical management. The rate of positive biopsies was 41%. All staff members and patients have accommodated to new adjustments. **Conclusion:** Activity in the breast unit dropped during the lockdown period. Staff should continue to seek their own and their patient’s safety without diminishing the quality of healthcare.

On 11 March 2020, the WHO classified COVID-19 as a pandemic [1]. Severe acute respiratory syndrome coronavirus 2 [2] belongs to the spectrum of viruses that cause the common cold, as well as more severe respiratory diseases – specifically, severe acute respiratory syndrome and Middle East respiratory syndrome, which have mortality rates of 10 and 34.4%, respectively [3,4]. COVID-19 was first identified in December 2019 [2]; its transmission occurs primarily through respiratory droplets from coughs or sneezes [5,6]. Its median incubation period is 5.1 days, and 97.5% of those who develop COVID-19 symptoms will do so within 11.5 days of infection [7]. All age groups are susceptible to the virus, especially elderly patients and those with comorbidities [8].

Lebanon, a Middle-Eastern developing country, confirmed its first case of COVID-19 on 21 February 2020 [9,10]. Despite the COVID-19 crisis, the radiology department, mainly the breast imaging unit, maintained its activity during this critical period.

To effectively reduce the risk of infection among radiologists and radiological technicians, a consensus on prevention and control measures in the radiology department was reached on 1 March, according to the recommendations of the Society of Breast Imaging [11]. For example, the secretary asked the patients to complete a questionnaire for potential exposure to COVID-19 before giving any appointment. Patient temperatures were taken before entering the radiology department, and patients were asked to wear a surgical mask. The staff followed the infection control guidance strictly when putting on and removing their protective equipment. A routine radiology equipment disinfection procedure was also established as wiping down surfaces between two patients. All machines, doorknobs, light switches, surfaces and handles were cleansed every 3–4 hours for staff and patient safety. Moreover, the hospital issued a statement demanding patients undergoing surgery or radiological interventions to be tested for COVID-19 with PCR by the end of April 2020.

The recommendations of the French Society of Breast Imaging were subsequently published on 7 May 2020 [12]. They included identifying patients at risk for having COVID-19, developing standard operating procedures for safe imaging of patients with suspected or known COVID-19, ensuring the availability of personal protective equipment (PPE) and educating healthcare workers, and implementing ‘social distancing’ strategies for staff, trainees and faculty members.

In this context, this study aims to describe the activity in the breast unit during this critical period and suggest how to accommodate during the post-lockdown period.

## Materials & methods

This was a retrospective descriptive study conducted among women who consulted the radiology department of a single university hospital institution (Hôtel-Dieu de France) during the COVID-19 lockdown period between March and May 2020. Lockdown was declared on March 9 and progressive un-lockdown during the first week of May.

For each patient, data on age, type of examination and final Breast Imaging-Reporting and Data System (BI-RADS) classification were collected. In case of biopsy, results were brought from the pathology department.

Mammograms were performed on a Selenia Dimensions System (Hologic^®^, Danbury, CT, USA). Each mammogram comprised bilateral craniocaudal and mediolateral oblique views. Supplemental views were done as needed. All ultrasound examinations were handheld and performed using a dedicated linear transducer with a frequency between 7 and 15 Mhz (Supersonic Imagine Aixplorer^®^, Aix-en-Provence, France) covering all breasts. Both color-Doppler and elastography were used when necessary.

A 3T MRI exam was performed using a dedicated 16-channel breast coil, covering both breasts (General Electric^®^, Wauwatisa, WI, USA). Patients who came twice or more to the department during the study time for biopsy, or for surgical needle localization, were counted once.

## Results

A total of 205 patients consulted the breast unit during the lockdown and immediately after lockdown week, with an equivalent of 264 visits. Patients’ mean age was 50.3 years (range 17–81 years).

**Figure 1.**
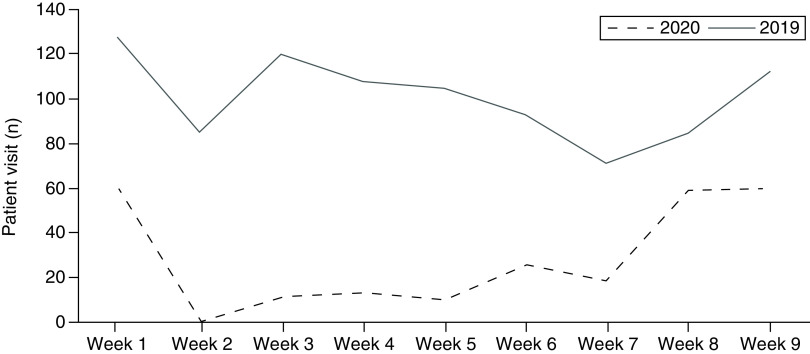
Distribution of patient visits over the period of lockdown in 2020 and comparison with the activity during the same period in 2019.

The number of patient visits over the study period and comparison with the activity in the same period of the previous year are represented in Table 1 and Figure 1.

**Table 1.** Distribution of patient visits over the period of lockdown in 2020 and comparison with the activity during the same period in 2019.

Date	Patient visit (n)	Date	Patient visit (n)
9–15 March	59	11–17 March	127
16–22 March	0	18–24 March	85
23–29 March	11	24–31 March	120
30 March–5 April	8	1–7 April	108
6–12 April	13	8–4 April	105
13–19 April	10	15–21 April	93
20–26 April	26	22–28 April	71
27 April–3 May	18	29 April–5 May	85
4–10 May	59	6–11 May	96
11–17 May	60	12–18 May	112

Some patients came more than once because of examinations scheduled on different dates. The total number of examinations was 153 mammograms, 205 ultrasounds, and 16 breast MRIs.

**Figure 2.**
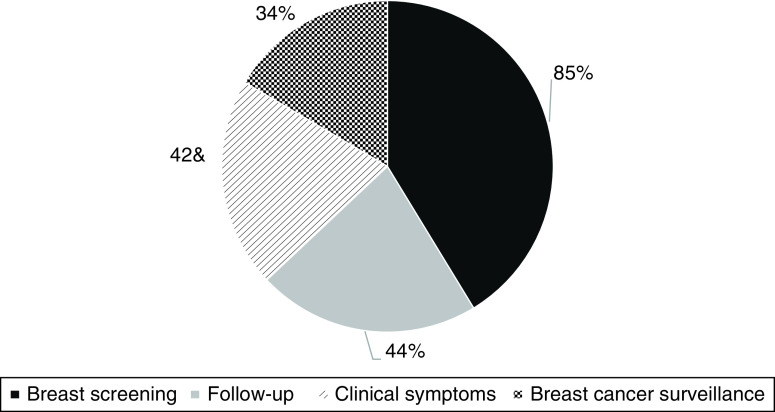
Indications for mammograms during the lockdown period.

Mammogram indications were breast screening (41.5%; 85 cases), breast cancer follow-up (22%; 45 cases), clinical symptoms (20%; 41 cases) and breast cancer surveillance (16.5%; 34 cases) (Figure 2).

Breast ultrasound was done to complement mostly mammography, such as dense breasts or in case of abnormality. It was also used for interventional guidance in all cases but two, where the biopsy was done under stereotactic guidance for a cluster of microcalcifications and under MRI guidance for lesions detected only on MRI.

Breast MRI was mostly done for preoperative surgical management (12 cases) and in case of radioclinical discordance (four cases).

Patient examinations were finally classified as BI-RADS 1 or 2 in 103 cases, BI-RADS 3 in 50 cases, BI-RADS 4 in 29 cases, BI-RADS 5 in 9 cases and BI-RADS 6 in 15 cases. BI-RADS 6 was reported in seven patients who had neoadjuvant breast cancer treatment and came for follow-up, and the others were presurgical localization. Among the BI-RADS four category, 25 patients already had a biopsy, and the four other cases had their biopsies scheduled during the study period. Five diagnoses of cancer were reported in this category. Among the BI-RADS 5 category, all 9 biopsied cases were reported to have cancer.

The rate of positive biopsies was 41% (14 out of 34): 13 cases of invasive cancers and one case of ductal carcinoma *in situ*. Thirty-two patients had biopsy under ultrasound guidance, one had biopsy under MRI guidance, and one under stereotactic guidance. The patient who underwent biopsy under MRI guidance had previous PCR. It was important to have a negative PCR test before undergoing an MRI examination because the cleaning of the MRI machine is difficult. The patient who had biopsy under stereotactic guidance did not have one, because no hospital recommendation was set at that time.

Eighteen patients required breast localization before their surgery. Only one patient received neoadjuvant chemotherapy. The rest of the patients were conservatively operated with an oncoplastic surgery. No patient turned out to be positive for COVID-19, and no radiologist or radiographer contracted COVID-19 during this period.

**Figure 3.**
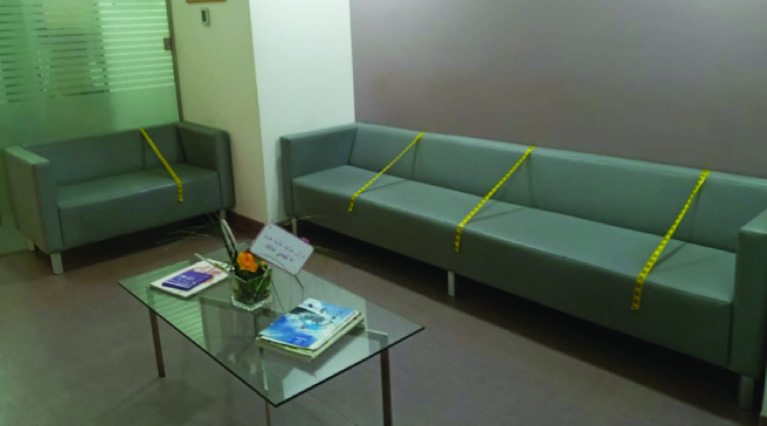
Adjustment of the waiting room in the breast unit.

**Figure 4.**
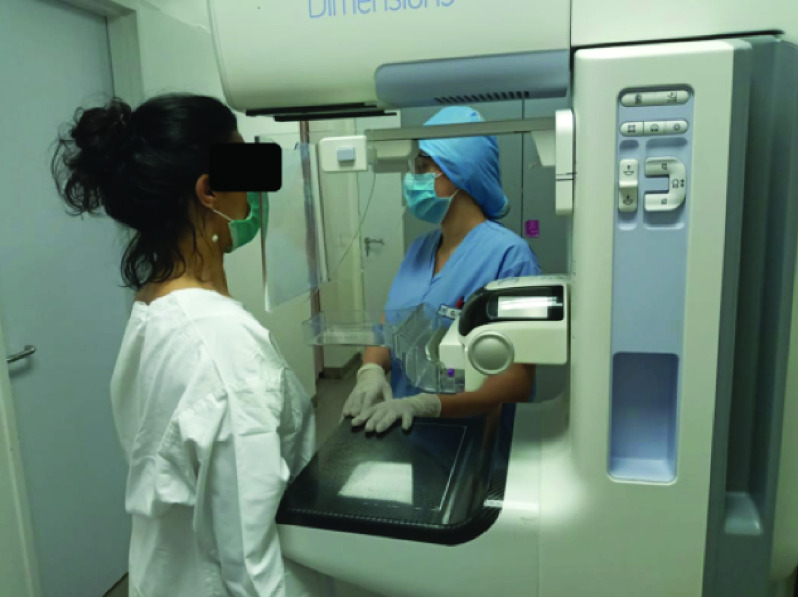
Radiology technician and patient wearing masks.

All staff members wore their PPE daily (Figure 3). The waiting room was adjusted (Figure 4), and patients were reassured by our new accommodations. Results were given immediately to the patients to avoid them having to come back to the unit. Weekly multidisciplinary staff, as well as teaching courses for the residents, were maintained online without interruption.

## Discussion

The COVID-19 crisis had a profound impact on breast unit activity in the radiology department, even in a country that did not attain a high-level incidence rate [13]. The number of visiting patients has fallen drastically during the lockdown period in comparison to the same period in the previous year. Contrary to some studies [12,14,15], mammograms, ultrasound or MRI examinations were not postponed or selected, but the indications for these examinations changed by themselves compared with the previous year. Fourteen new cases of breast cancer were detected among 205 patients who consulted our unit. All but one were invasive breast carcinomas.

The activity in the breast unit may increase rapidly and immediately after the lockdown period to quickly reach its level before the crisis, in case of no restrictions on appointments. No restriction on screening mammograms immediately after the lockdown was suggested in a recent study [12]. To our astonishment, patients overcame their apprehension to consult and were reassured by the staff PPE and their cleansing procedures.

The novelty of this article is the continuity of screening during the pandemic, enabling us to prevent delaying diagnoses and consequently treating cancer patients, which might lead to upstaging and poor prognosis. In addition, pursuing our screening and diagnostic procedures did not expose the patients to higher risk of COVID infection. In fact, as suggested by colleagues in a new published article, breast cancer patients may not be at higher risk of developing a deadly COVID infection [16].

Therefore, until the COVID-19 crisis ends, it is crucial to continue in preventing the transmission of the virus to patients and radiology department staff members by pursuing all previously issued recommendations. For patients, wearing a mask and spending the shortest time possible in the radiology department are mandatory. This could be achieved by giving the patients online appointments, arranging paperwork ahead of time, asking them not to be accompanied, making the transition from mammography to ultrasound as quickly as possible and giving them results immediately. For the staff, using personal protective devices should continue.

PCR before every interventional breast biopsy was discussed among all radiologists in our department and with the department of gynecology since no society recommendations were available until now. To the best of our knowledge, this is the first publication that discusses the need for PCR before breast interventional procedures. In our opinion, PCR testing before any ultrasound-guided breast biopsy is questionable. However, it seems to be indicated in case of ultrasound-guided biopsy for BI-RADS 5 lesions because the patient will need it definitely before surgery, and it could help to get a breast MRI examination. In addition, during the interventional procedures, the staff will have contact with the patient for longer than 15 minutes and with a distance less than 1.5 m, which would make the safety of the involved staff questionable and expose them to risk of infection in case of unknown COVID status of the patient. PCR also seems mandatory before biopsy under MRI or stereotactic guidance because of the long duration of these examinations, the difficulty in cleaning the MRI unit and the proximity of staff to the patient. Finally, the weekly multidisciplinary staff and teaching courses for the residents should be maintained online without interruption.

## Conclusion

Activity in the breast unit dropped during the lockdown period as expected, but it increased quickly and immediately after. Staff should continue to seek their own and their patient’s protection without diminishing the quality of healthcare. PCR seems mandatory before biopsy of lesions under MRI or stereotactic guidance. Scientific activities can be maintained online.

**Summary points**

- The activity of the radiology department of a breast unit in a Lebanese tertiary center dropped during the coronavirus disease 2019 lockdown.
- The main indication for mammogram during this coronavirus disease 2019 period was breast cancer screening.
- MRI was performed mainly for preoperative surgical management.
- Of the performed breast biopsies, 41% were positive.
- PCR testing was performed before any biopsy or MRI examination.
- All staff wore personal protective equipment, and distance adjustments were made to the waiting area.
- No positive cases were noted among patients or staff.
- Weekly multidisciplinary staff and teaching courses for the residents were maintained online without interruption.
